# Effects of different management regimes on microbial biodiversity in vineyard soils

**DOI:** 10.1038/s41598-018-27743-0

**Published:** 2018-06-20

**Authors:** Maximilian Hendgen, Björn Hoppe, Johanna Döring, Matthias Friedel, Randolf Kauer, Matthias Frisch, Andreas Dahl, Harald Kellner

**Affiliations:** 1 Department of Soil Science and Plant Nutrition, Hochschule Geisenheim University, Geisenheim, Germany; 2 Department of General and Organic Viticulture, Hochschule Geisenheim University, Geisenheim, Germany; 3 Department of Soil Ecology, Helmholtz Centre for Environmental Research – UFZ, Halle, Germany; 4 Institute for National and International Plant Health, Julius Kühn-Institute, Braunschweig, Germany; 5 Institute of Agronomy and Plant Breeding, Justus Liebig University, Gießen, Germany; 6 Deep Sequencing Group - Biotechnology Center Technische Universität Dresden, Dresden, Germany; 7 Department of Bio- and Environmental Sciences, International Institute Zittau, Technische Universität Dresden, Dresden, Germany

## Abstract

An active and diverse soil biota is important for maintaining crop productivity and quality, and preservation of these traits is a major goal of sustainable farming. This study aimed at unravelling the impact of different management practices on soil fungal and bacterial biodiversity in vineyards as a model for permanent crops. Species diversity was assessed using an amplicon sequencing approach in a long-term field experiment in the Rheingau wine region of Germany where integrated, organic and biodynamic management practices had been in place for 10 years. Fungal community composition under integrated management differed significantly from organic and biodynamic management, whereas fungal species richness remained unaffected. Soil under integrated management had a significantly reduced bacterial species richness compared to organic, but community composition was similar to organically and biodynamically managed soils. Highest fungal richness was obtained under cover crop between rows in topsoil, arising from cover cropping and organic carbon supply.

## Introduction

The microbiome of a soil impacts on organic matter decomposition^1^, nutrient cycling and buffering^2^, soil structure^3^, redox balance^2^ and the degradation of pollutants^4^. Furthermore, it influences plant health and growth through positive benefits for processes such as or mycorrhization^5^, symbiotic interaction^6^ and resistance induction^7^, or negatively through pathogenic infection^8^. Thus, the microbiota can be considered as a key player in soil functionality, ensuring soil productivity and product quality in agricultural production systems^9^. Despite the fact that little is known about the importance of single organisms for ecosystem functionality and about redundancy between organisms^10^, it is commonly agreed upon that high biodiversity can ensure vital and productive soils and buffers negative impacts^11–13^.

In viticulture, soils can be subjected to intensive cultivation over long-term time scales as part of cover cropping, soil water and weed management practices. Viticulture soils also receive frequent application of herbicides and fertilizers, and may accumulate copper or other fungicides following foliar applications for disease control^14,15^. However, integrated management may differ substantially from organic and biodynamic management systems concerning cultivation practices and chemical inputs, and this may influence the vitality and composition of soil organisms. Furthermore, vineyard sites are often established on less fertile and shallow soils, characterized by low water holding capacity, high sun exposure and low nitrogen availability, and are therefore regarded as sensitive ecosystems. Hence, viticulture in general, and integrated farming in particular, is often considered to reduce soil biodiversity and to drive soil biota extinction by the use of soil-destructive farming practices^16^, resulting in depleted grounds^17^. Nevertheless, vineyard soils are of major interest as they are an important determinant of grape and wine quality, a common basis for the classification of vineyard sites and part of the so called “terroir” of wines^18,19^.

In agriculture, organic farming is a constantly booming sector, exhibiting a total area of 57.8 million hectares worldwide and a growth rate of 420% since 1999 (standing 2016)^20^. Grape production is following this trend as well; comprising 380,000 ha worldwide that account for 5.3% of the total crop area, and of which 90% are located in Europe^20^. This rapid increase of organically farmed land, driven by political will and consumer demand, has sparked increasing scientific interest in the comparison of farming systems. Most studies focused on soil fertility, yield, crop quality and plant physiology; however, results are somewhat inconsistent^21^. So far, publications about the impact of management regimes on soil microbial diversity are only available for arable farming^22,23^, whereas results for viticultural land use are lacking. In this respect, traditional cultivation methods and phospholipid fatty acid (PLFA) profiling face a range of difficulties in evaluating the complete diversity of soil microflora^24^, as substantial amounts of microbial organisms and their ecology still need to be investigated. In addition, former studies on viticulture mostly focused on the impact of a single factor like copper contamination, or were conducted under artificial circumstances, on a small soil scale or in a short time frame^25–27^. Therefore, interaction effects like within the combined application of pesticides and herbicides or long-term effects like extinction debt could scarcely be detected^16^. To overcome these constraints, and to improve future agricultural management towards the preservation of edaphic biodiversity, the goals of this study were

1. to explore differences in fungal and bacterial populations between soils under integrated, organic and biodynamic vineyard management in a long-term field experiment,
2. to gain novel insights into the microbial biodiversity in a long-term vineyard soil in the Rheingau region in Western Germany, and
3. to relate differences of the microbiome among management systems to specific management practices and resulting soil factors (in order to improve the sustainability of vineyard management).

To reach these goals, soil samples were taken at a long-term field trial, and biodiversity of soil fungi and bacteria was determined using a barcoding approach^28–30^. Furthermore, chemical and physical parameters of the soil samples were examined to screen for correlations with fungal and bacterial richness and community composition.

## Results

### Soil analysis

Altogether, soil analyses revealed largely homogenous ground conditions (Supplementary Table 1). However, some minor yet significant differences were detected according to the three investigated factors: Soil organic carbon, soil moisture content and potassium varied significantly in relation to management practice, position and depth (α = 0.05). With an average value of 1.25%, soil organic carbon content was highest in in-row topsoil, whereas values for subsoil samples were decreased by about 0.3%. Soil moisture content was rather low due to a previous drought period (~8.5%) with slightly lower values in topsoil compared to subsoil and in-row compared to under-vine (Supplementary Fig. 1). The only parameter exhibiting distinct differences solely according to the management system was magnesium; however, mean values ranged only between 12.81 mg 100 g^−1^ (org = highest) and 10.75 mg 100 g^−1^ soil (biodyn = lowest). Nitrogen (mean 0.086%) and pH (mean 7.35) differed slightly in comparison of top- and subsoil (Supplementary Fig. 1). Besides this, soil from the biodynamical farmed parcels showed increased total amounts of the heavy metals iron (about 23.8 g kg^−1^ vs. ~22.5 g kg^−1^), manganese (about 700 mg kg^−1^ vs. ~628 mg kg^−1^) and zinc (about 105 mg kg^−1^ vs. ~95 mg kg^−1^) compared to the integrated and organic farming.

### Species richness

**Figure 1:**
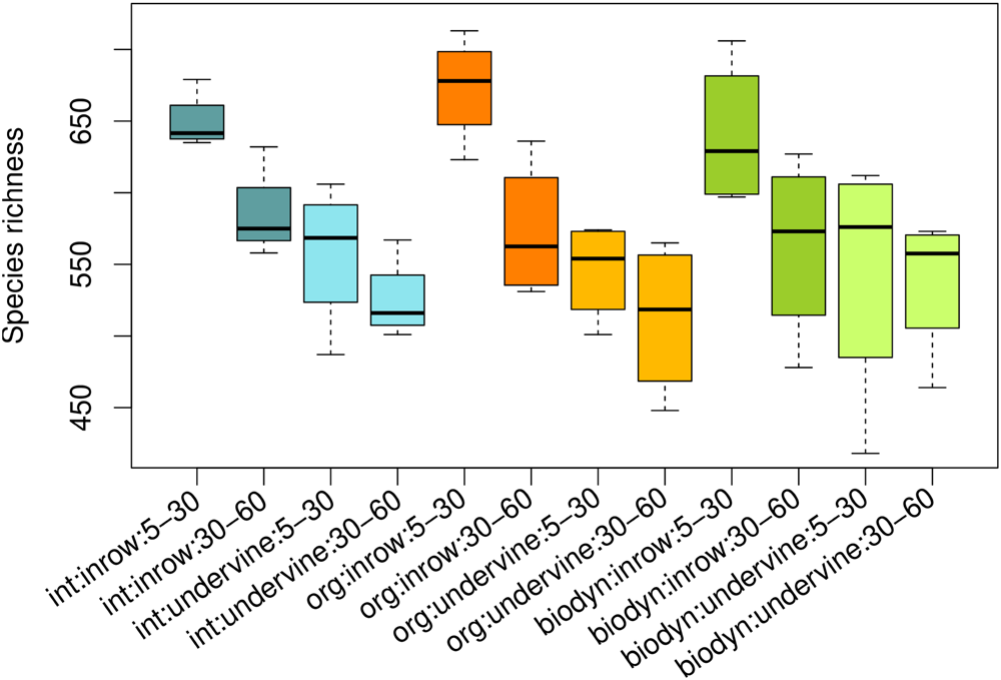
Fungal species richness by management, position and depth. Levels of significance: p (management) <0.819, p (position) <6. 36E-4, p (depth) <0.0003. F-values: F (management) = 0.200, F (position) = 38.231, F (depth) = 16.319. Degrees of freedom: DF (management) = 2, DF (position) = 1, DF (depth) = 1 (n = 4).

The normalized fungal data set consisted of 988,099 sequences overall, which were clustered into 2,309 *operational taxonomic units* (OTUs). Fungal richness ranged between 418 and 713 species per sample (Fig. 1). Mean species richness was 579 for integrated, 576 for organic and 571 for biodynamic practice. The analysis of variance revealed no significant effect of the management regime (α = 0.05), whereas depth and position did have significant effects (both p < 0.001) with a higher fungal species richness in topsoil (mean 601 vs. 548 in subsoil) and in-row (mean 615 vs. 537 in under-vine), respectively (Fig. 1).

**Figure 2:**
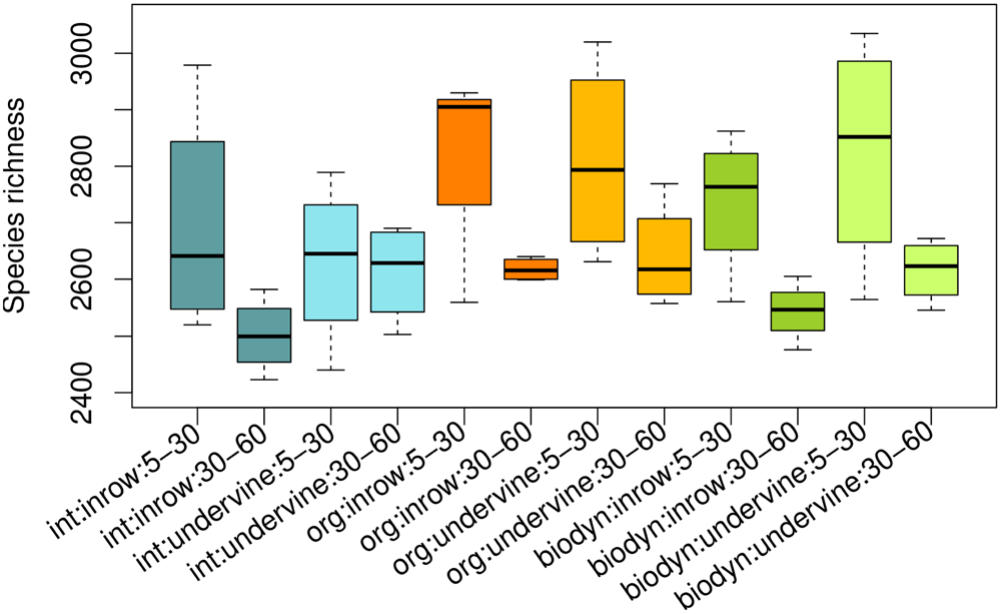
Bacterial species richness by management, position and depth. Levels of significance: p (management) <0.020, p (position) <0.266, p (depth) <9.20E-6 F-values: F (management) = 4.387, F (position) = 1.282, F (depth) = 27.414. Degrees of freedom: DF (management) = 2, DF (position) = 1, DF (depth) = 1 (n = 4).

The normalized bacterial data set was composed of 1,193,925 reads, which were clustered into 8,254 OTUs. Bacterial richness varied from 2,423 to 3,035 species per sample (Fig. 2). According to the analysis of variance, soil depth had the biggest influence on bacterial species richness (p < 0.001) with a higher richness in topsoil (mean 2,753 vs. 2,588 in subsoil). Further, a significant effect (p < 0.05) was also revealed for management practice, with 2,609 bacterial species occurring under the integrated management, 2,723 under organic, and 2,680 under the biodynamic farming system. This represented an increase of 4.4 and 2.7% respectively, for the two treatments. Pairwise comparison of the management systems however solely showed a significant difference between integrated and organic management (p < 0.018). Biodynamic and integrated management did not differ substantially (p < 0.114), nor did organic vs. biodynamic treatment (p < 0.280). Position had no effect, with a mean in-row value of 2,653, and mean under-vine value of 2,688 species per sample.

### Species community

Based on the results of the *permutational multivariate analysis of variance* (PERMANOVA), management regime, position as well as depth significantly affected the fungal community composition. Interactions did not occur at α = 0.05 (Table 1).

**Table 1:** PERMANOVA of fungal species communities.

	Df	SumsofSqs	MeanSqs	F.Model	R^2^	Pr(>F)
management	2	0.5581	0.27904	2.4649	0.07959	0.001^***^
position	1	0.6611	0.66109	5.8395	0.09428	0.001^***^
depth	1	1.0096	1.00957	8.9117	0.14397	0.001^***^
management:position	2	0.2563	0.12817	1.1321	0.03655	0.245
management:depth	2	0.1844	0.09218	0.8142	0.02629	0.808
position:depth	1	0.1615	0.16145	1.4261	0.02302	0.091
management:position:depth	2	0.2191	0.10955	0.9677	0.03124	0.494
Residuals	35	3.9623	0.11321		0.56505	
Total	46	7.0123			1.00000	

Significance code: [.] <α = 0.1; [*] < α = 0.05; [^**^] <α = 0.01; [***] <α = 0.001.

**Figure 3:**
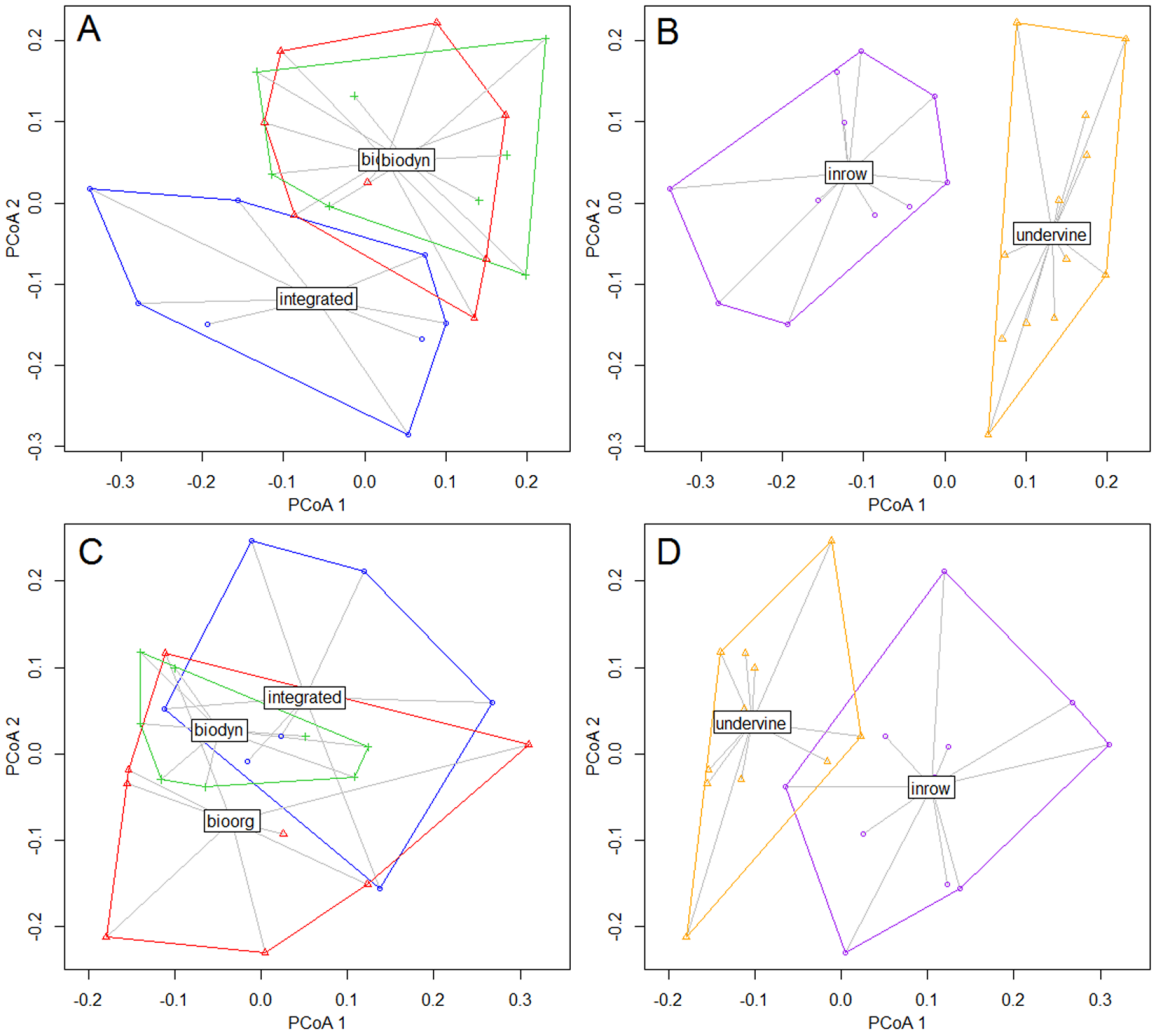
Fungal species community according to (**A**) management in topsoil, (**B**) position in topsoil, (**C**) management in subsoil and (**D**) position in subsoil. PCoA displaying group centroids and dispersions.

Coefficients of determination attributed 14.6% of community differences to soil depth, 9.5% to position and 7.7% to the management regime. Overall, the three factors were responsible for 43.5% of community differences, whereas 56.5% could not be explained through this model. Subsequently performed *Priciple Coordinates Analysis* (PCoA) ordinations revealed considerable differences according to the management regime, with integrated fungal community diverging from organic and biodynamic, the latter both highly similar to each other (Fig. 3A,C). *Analysis of similarities* (ANOSIM) proofed these findings, displaying a significant difference between the integrated and the organic management (p < 0.023) as well as between the integrated and the biodynamic management (p < 0.027). Fungal communities of the organic and biodynamic treatment were statistically not distinguishable from each other (p < 0.810). The delimitation of the fungal community under integrated management was stronger in topsoil (p < 0.001) compared to subsoil (p < 0.026), whereas the community differences according to in-row and under-vine position were consistent in both top- and subsoil (p < 0.001) (Fig. 3B,D). Further PERMANOVA analyses with data subsets confirmed the depth-dependent management effect and revealed the in-row topsoil as core area of community distinctions by management (p (management) < 0.005 for inrow-topsoil compared to p (management) <0.076 for undervine-topsoil; Supplementary Tables 2 to 5), indicating a strong relevance of cover crop composition and their correspondent rhizospheres on soil-borne fungi.

Bacterial species communities differed highly significantly between top- and subsoil, with an R squared of 13.9%. Less strong, but as well significant were the effects of in-row and under-vine position, but with an R squared of 4.3% position impacted only weak on bacterial community differences. Management regime showed a significant effect as well, yet the p-value < 0.022 designates management to be less affecting in comparison to the fungal community results (Table 2).

**Table 2:** PERMANOVA of bacterial species communities.

	Df	SumsofSqs	MeanSqs	F.Model	R^2^	Pr(>F)
management	2	0.10166	0.050829	1.3871	0.05087	0.022^*^
position	1	0.08617	0.086169	2.3515	0.04312	0.004^**^
depth	1	0.27761	0.277614	7.5758	0.13892	0.001^***^
management:position	2	0.0593	0.029651	0.8091	0.02967	0.536
management:depth	2	0.06622	0.033111	0.9036	0.03314	0.367
position:depth	1	0.03316	0.033156	0.9048	0.01659	0.32
management:position:depth	2	0.05505	0.027527	0.7512	0.02755	0.701
Residuals	36	1.31922	0.036645		0.66014	
Total	47	1.9984			1.00000	

Significance code: [.] <α = 0.1; [^*^] <α = 0.05; [^**^] < α = 0.01; [^***^] <α = 0.001.

**Figure 4:**
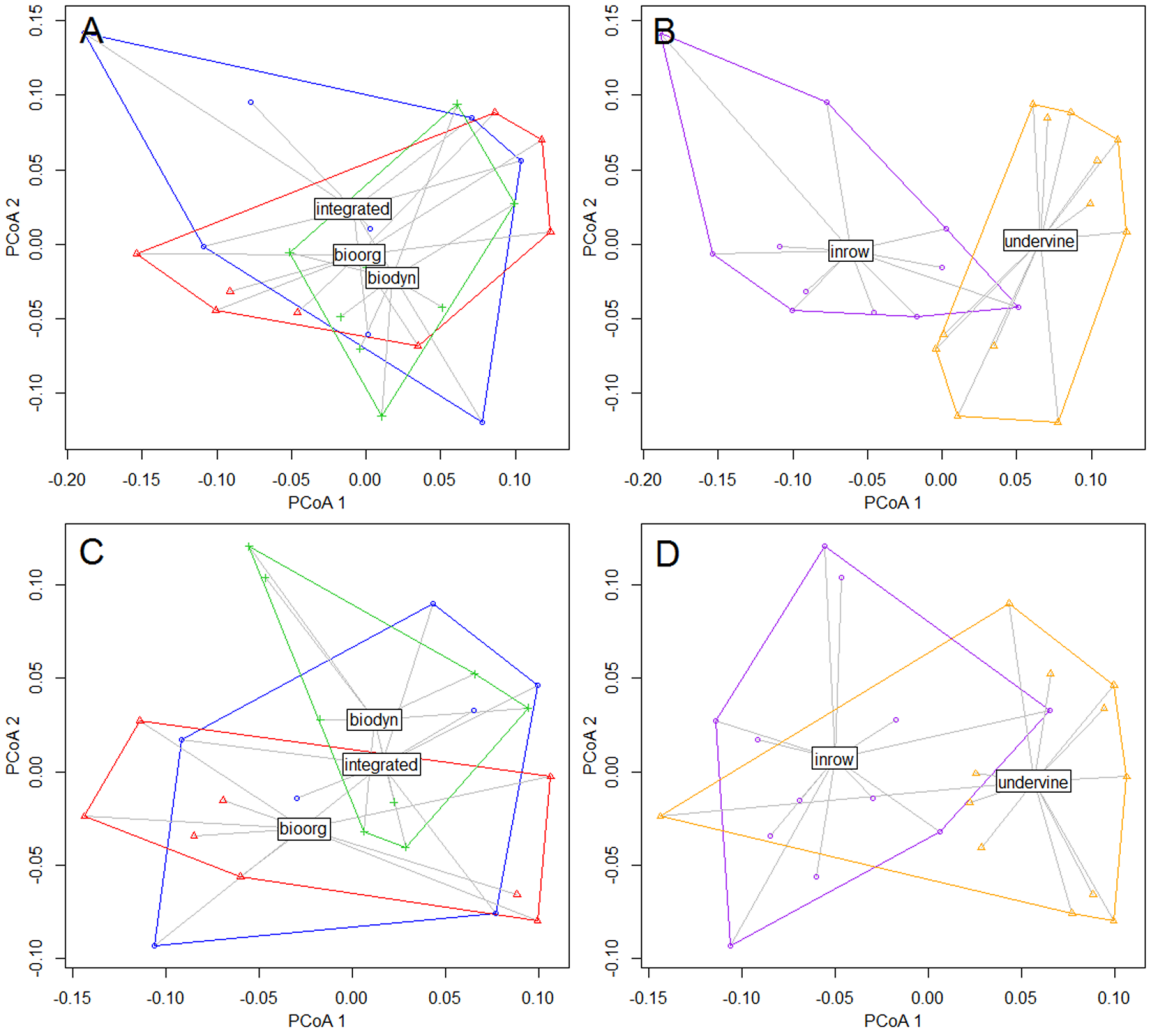
Bacterial species community according to (**A**) management in topsoil, (**B**) position in topsoil, (**C**) management in subsoil and (**D**) position in subsoil. PCoA displaying group centroids and dispersions.

PCoA ordinations affirmed these findings, showing a high grade of conformity between bacterial communities under the three management regimes in top- as well as in subsoil (Fig. 4A,C). ANOSIM nonetheless revealed a significant difference between the integrated and the organic management (p < 0.013) as well as between the integrated and the biodynamic management (p < 0.001) in topsoil, whereas the organic and biodynamic management showed no statistical difference (p < 0.713). For subsoil in contrast, the ANOSIM exhibited no significant differences between any of the three management systems. For in-row compared to under-vine position, the PCoA ordinations implied a higher grade of dissimilarity in topsoil than in subsoil (Fig. 4B,D). A total of 66% of the community variance could not be explained by the three reviewed main factors.

Analysis of correlation identified organic carbon, pH, nitrogen, and potassium as strongest determinants (p < 0.001) of fungal community structure. Minor influence was attributed to soil moisture content and bioavailable iron (p < 0.01). For the bacterial community, the best correlation was found for organic carbon and potassium (p < 0.001), followed by nitrogen and bioavailable copper (p < 0.01). A weak relation was attributed to pH, magnesium, total manganese and zinc (p < 0.05). Detailed results of the correlation analysis for fungi and bacteria can be reviewed in the Supplementary Tables 6 and 7.

### Taxonomic classification

**Figure 5:**
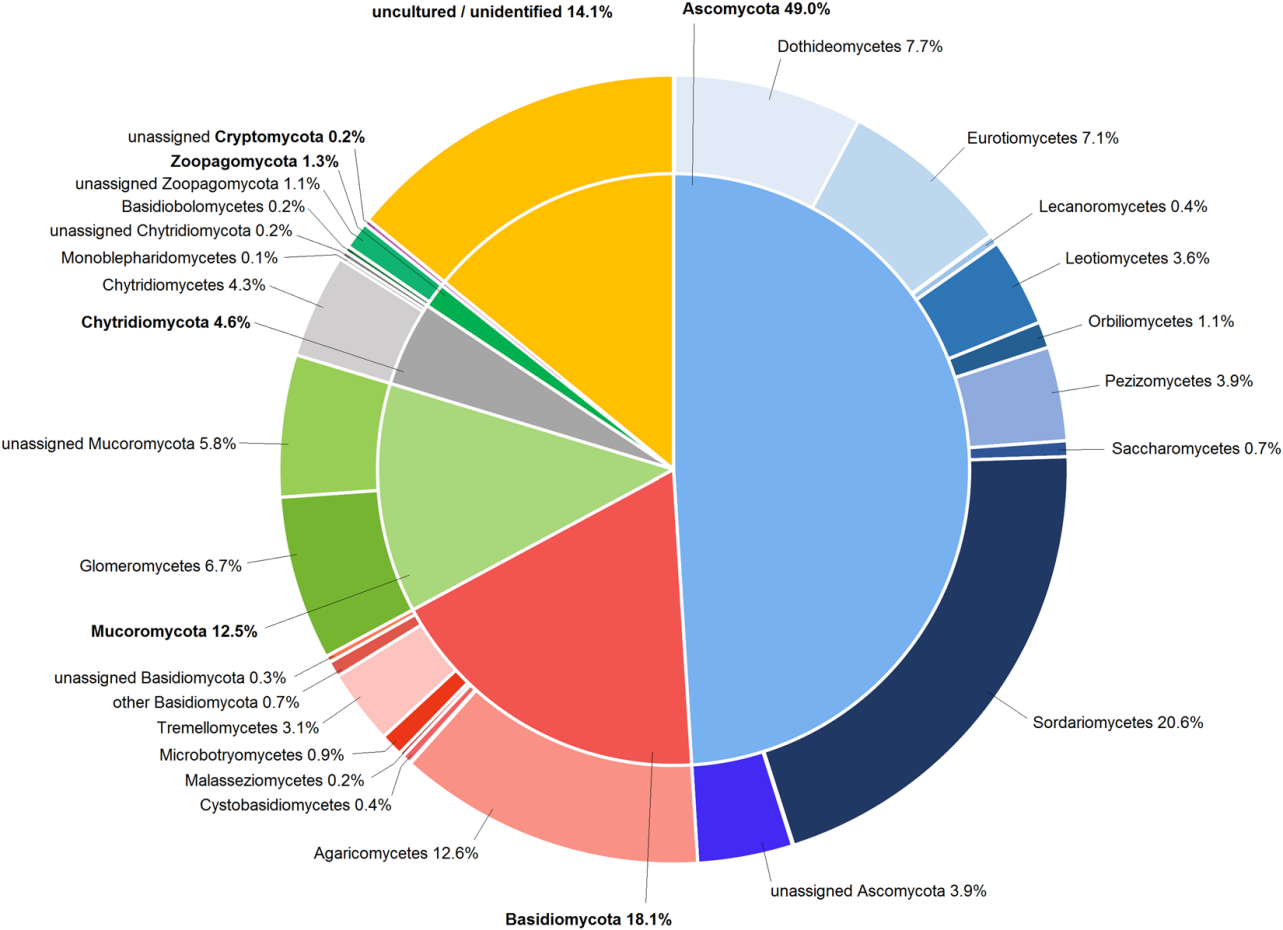
Proportions of fungal phyla and classes of the whole data set based on OTUs [%].

**Figure 6:**
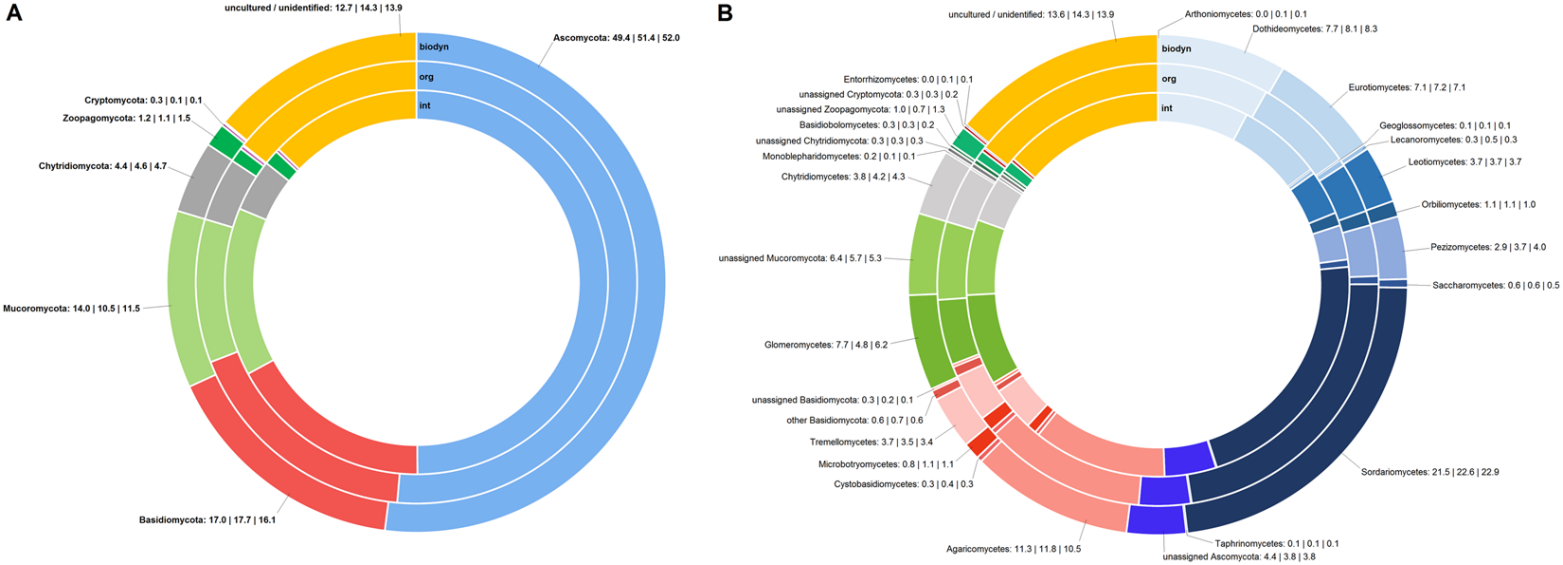
Proportions of (**A**) fungal phyla and (**B**) fungal classes according to management in topsoil [%] based on OTUs. For fungal classes, proportions are listed behind the denomination in the order int | org | biodyn in %. For topsoil, richness showed no significant differences according to management as well (richness means: int = 603, org = 609, biodyn = 592).

Referencing the representative sequences of all 2,309 fungal OTUs against a database led to their attribution into six phyla, 34 classes and 249 families. Among the six phyla, one was less than 1% abundant (Cryptomycota: 0.2%). 14.1% of the sequences were assigned “uncultured” or “unidentified”. On a OTU basis, the affiliation to Ascomycota was highest (49.0%), followed by Basidiomycota (18.1%) and Mucoromycota (12.5%) (Figs 5 and 6). In comparison, sequence-based evaluation of abundance levels revealed proportions of 77.4% Ascomycota, 14.0% Basidiomycota and 6.0% Mucoromycota (results not shown).

For further investigation of the fungal community distinctions according to management, phylum and class proportions were investigated for topsoil and according to the three systems (Fig. 6A,B). The integrated system showed a slightly reduced proportion of Ascomycota (49.4%) compared to organic (51.4%) and biodynamic (52.0%) management (Fig. 6A). On the other hand, Mucoromycota showed higher proportions in the integrated system (14.0 vs. 10.5 and 11.5%). Proportions of Basidiomycota were highest for the organic management (17.7%). The less abundant phyla did not vary substantially according to management.

Regarding the class proportions (Fig. 6B), none of the different phyla ratios was based on a single class. The reduced ratio of Ascomycota of the integrated management mostly based on combined smaller proportions of Dothideomycetes (7.7 vs. 8.1 and 8.3%), Pezizomycetes (2.9 vs. 3.7 and 4.0%) and Sordariomycetes (21.5 vs. 22.6 and 22.9%). On the other hand, the higher ratio of Mucoromycota based on both Glomeromycetes (7.7 vs. 4.8 and 6.2%) and unassigned Mucoromycota (6.4 vs. 5.7 and 5.3%). Proportions of Saccharomycetes which play an important role in grape must fermentation were of similar size in all three management systems (0.6 vs. 0.6 and 0.5%). Arthoniomycetes and Entorrhizomycetes were solely present in soils of the organic and biodynamic management but were both of minimal abundance (0.1%).

The 30 fungal OTUs with the highest abundance aggregated to approximately 50% of all sequences, underlining their importance as stable “core OTUs” over all samples. The most abundant fungus was identified as *Solicoccozyma (Cryptococcus) aeria*, a yeast that belongs to Basidiomycota, followed by a *Fusarium* sp. and a *Verticillium* sp., both belonging to Ascomycota (Supplementary Fig. 2).

**Figure 7:**
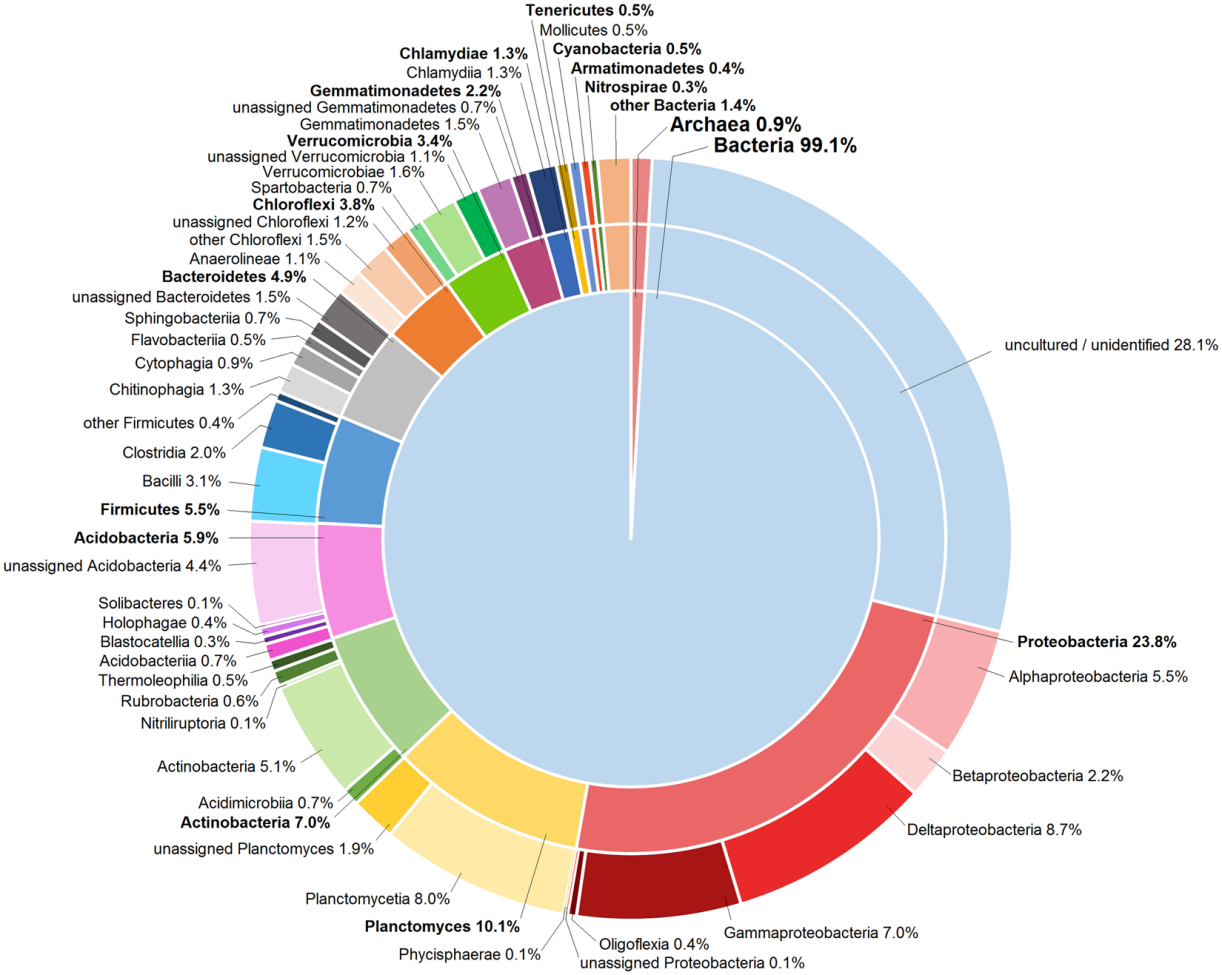
Proportions of bacterial phyla on the whole data set based on OTUs [%].

For bacteria, the database query of representative 16S rRNA sequences revealed an affiliation of 99.1% of OTUs to bacteria and 0.9% to archaea. Among the bacteria and archaea, 2316 sequences were marked “uncultured” and “unidentified”, which made up 28.1% of all OTUs. The remaining 5938 OTUs could be assigned to 32 different phyla, with ten major phyla reflecting over 96% of all assigned bacteria (Fig. 7). The most prominent phyla Proteobacteria (23.8%) accounted for one third of all assigned bacterial OTUs, followed by Planctomycetes (10.1%) and Actinobacteria (7.0%). Proportions of Acidobacteria Firmicutes, Bacteroidetes, Chloroflexi, Verrucomicrobia, Gemmatimonadetes, and Chlamydiae ranged from 5.9–1.3%. The remaining 18 phyla summed up to 2.9%. On class level, the 5938 OTUs were assigned to 79 different classes, of which the 29 most abundant ones are displayed in Fig. 7.

The 30 bacterial OTUs with the highest abundance (Supplementary Fig. 3) incorporated about 27% of all bacterial sequences, and were composed of two Archaea and seven bacterial phyla. The most abundant OTU was identified as *Nitrosphaeria viennennsis* belonging to the domain of Archaea, followed by *Aridibacter kavangonensis* and an uncultured bacterium.

## Discussion

In this study, we present a first comprehensive overview of the fungal and bacterial communities in a vineyard soil under different management practices based in the Rheingau wine region in Germany. The physical and chemical soil parameters were considered representative of vineyard soils in this region, and the soil nutrient status was sufficient with regard to grape production^31^. Soil heavy metal concentrations were also below levels of phytotoxicity^32,33^. Ten years of different management regimes did not lead to substantial changes between the vineyard treatments. However, some significant effects, e.g. in soil moisture content as consequence of cover crop rooting depth, could be attributed to the differences in soil management. In relation to other surveys on vineyard soil microbiota, which have around 100–200 fungal OTUs^34,35^, our fungal species richness of about 400–700 species turned out to be higher. But for the comparison it has to be kept in mind that sampling depth in this study was two- to threefold deeper, and the sequence data coverage was more extensive due to the modern barcoding method. The decrease of fungal species richness with increasing soil depth is consistent with literature and presumably due to the decrease in organic carbon and nitrogen^2,36^. Nevertheless, the mean species richness in subsoil reached about 90% of the topsoil mean values. In-row topsoil layer proved to be highest in the here analyzed fungal species richness, which underlines a positive impact of cover crop vegetation on soil-borne fungi. The rhizosphere under in-row vegetation provides a biotope with favorable conditions, and root exudates might promote the microbial diversity by being a steady source of organic carbon^37,38^. Additionally, mowing the biomass brings a regular input of organic matter into the soil. Bare under-vine strips lacked such benefits; however, there was not any difference between tillage and herbicide treatment.

The fungal richness was not influenced by different management practices, whereas a distinct change of the fungal community was observed between integrated and organic/biodynamic practice in the topsoil. To our knowledge, this is the first evidence for such management effects in a perennial crop. However, in general, this is consistent with the findings of Hartmann and Widmer^23^ for soils in crop rotation systems. As this fungal community shift is most obvious in topsoil from between the vine rows, it may be related to the choice of cover crop which can influence microbial communities by dint of plant-specific root exsudates, litter composition, soil aggregation and moisture^39^. This possibly can provide a tool for winegrowers to manage the fungal community to some extent. Furthermore, we show that the additional input of biodynamic preparations did not affect the fungal composition or richness compared to the organic treatment.

Spatial patterns of fungal communities according to depth and position underlined the occurrence of local biotope subdivisions in vineyard soil due to periodic under-vine tillage. The slightly reduced relative amount of Ascomycota under integrated management might be caused by the use of systemic fungicides, which might act more specifically against Ascomycota than copper and sulfur, which are applied in organic/biodynamic treatments. A beneficial effect of the increased ratio of Glomeromycetes in the integrated management cannot be attributed as amplicon sequencing studies lack the taxonomic accuracy to identify *Vitis vinifera*-specific mycorrhiza species.

Interestingly, community distinctions due to management only manifested in topsoil, whereas those due to position were depth-independent. As the whole site was deep ripped about 60 cm deep before planting in 1991 and therefore homogenized in soil, it can be assumed that ten years of different management did not suffice to induce differences in subsoil communities, whereas 24 years since planting sufficed to establish distinct in-row and under-vine habitats in top- and subsoil. Furthermore, the ten years of different management practices neither brought major shifts in physicochemical soil parameter, nor sufficed to affect the most abundant fungi in this probed vineyard soil. According to this, the community distinctions between the integrated, organic and biodynamic treatments related to less abundant OTUs, whereas the predominant ones generated a set of core-OTUs with ubiquitous distribution^35^.

Bacterial richness exceeded the fungal one, reflecting the generally higher bacterial diversity and occurrence^40,41^. Our values ranging from 2,423 to 3,035 OTUs per sample were comparable to results of other publications, which report bacterial species richness between 2,768 and 3,664 OTUs from vineyard soils^42^. Bacterial species richness showed the same depth-related trend as the fungal richness, following the gradients of organic carbon and nitrogen as well as decreasing gas and water exchange. Conversely, position seemed not to affect bacterial species richness, and neither cover cropping nor tillage exhibited a positive or negative impact on bacterial biodiversity. Topsoil samples merely showed a higher variance than subsoil samples, indicating higher spatial heterogeneity closer to the soil surface. In contrast to the fungal findings, organic and biodynamic farming seemed to yield higher bacterial richness, though the effect was weak and statistically significant solely in the organic treatment. Possible explanations could be mutual relationships between some bacteria and the diverse herbaceous cover cropping^43^, or a positive impact of the compost fertilizer^44,45^. The varying bacterial species richness did also transfer into distinctions between species communities, with the bacterial community under integrated management differing from those under organic and biodynamic farming. However, this distinction was only detectable in topsoil, and the higher p-value as well as the PCoA ordination revealed a weaker management effect compared to the fungal findings. Accordant to the fungal result, bacterial community composition exhibited the strongest changes following soil depth^46,47^. Results showed that position had some influence as well, but community differences were less distinct compared to fungi. There was a high portion of OTUs belonging to a stable community core present in all samples as visible in community ordination, analogous to Zarranoindia *et al*.^43^. Hence, the bacterial community can be assumed to be rather insensitive against influences exerted by the three management systems. However, as DNA-based richness scores do not provide any information about metabolic activity and as databases mostly lack ecological knowledge for bacterial hits it is vague to evaluate the functional importance of individual present or absent OTUs. Bacterial phyla composition and proportions mostly followed taxonomic community patterns similar to those revealed by other groups^43,48,49^, thus indicating high consistency of bacterial communities over several locations and climates, mostly affected by land use patterns. Solely proportions of Planctomycetes tended to be higher in our study; whereas the ratio of Acidobacteria was smaller instead. However, as an association of these two phyla with grapevines has not been reported yet, its relevance for viticulture has to remain unanswered.

Future studies dealing with management impacts on soil microbiota might upgrade their explanatory model by including also soil physical parameters and detailed vegetation assessments, as cover crops showed to be a major determinant of species richness and composition in our permanent culture. In addition, temporally expanded surveys using RNA sequencing over one growing season might help to gain knowledge about activity patterns of similar communities.

## Method

### Experimental site

The field experiment was conducted in Geisenheim, Germany (49°59′22.0′′N 7°57′00.7′′E). The experimental site was 0.8 hectare in size (*Vitis vinifera* L. cv. Riesling, clone Gm 198–30, grafted on *Vitis berlandieri* Planch. x *Vitis riparia* Michx. cv. SO4 and *Vitis riparia* Michx. x *Vitis cinerea* Engelm. cv. Börner rootstock, respectively). It was planted in 1991 and conversion to organic and biodynamic viticulture started in 2006. The vines were planted at a spacing of 1.2 m within rows and 2 m between rows using a vertical shoot positioning system (VSP). Until the end of 2005 the vineyard was managed according to the code of good practice^21,50^.

The experiment was set up as a complete block design, in which the three factor levels of the main effect management system were replicated in four blocks. Each plot consisted of four rows with 32 vines each (Supplementary Fig. 4). Only the inner two rows of each plot were used for data collection. The plots were checked for uniformity prior to data collection in 2010 concerning physical and chemical soil parameters^21^. Long term annual rainfall for the site is 543 mm^51^. Total rainfall in 2015 was 396 mm and growing season rainfall was 227 mm^51^.

The integrated treatment was managed according to the code of good practice^50^. Organic and biodynamic plots were managed according to Regulation (EC) No 834/2007^52^ and Regulation (EC) No 889/2008^53^ and according to ECOVIN and Demeter standards, respectively (Table 3).

**Table 3:** Distinctions in management practices among integrated, organic and biodynamic treatment.

Management practice	integrated	organic	biodynamic
cover crop	grass mixture	Wolff mixture	
under-vine management	herbicides	mechanically	
fertilization	mineral fertilizers + compost	plowing in cover crop + compost	plowing in cover crop + compost with biodynamic preparations
plant protection	systemic fungicides mating disruption	copper + sulphur + plant resistant improvers mating disruption	
biodynamic preparations	—	—	horn manure, horn silica, compost preparations

(modified according to Döring *et al*.^21^).

All three treatments periodically received compost. Green waste compost was used for the integrated plots and farmyard manure for the organic and biodynamic plots. In addition, the biodynamic compost preparations of *Achillea mellefolium*, *Matricaria chamomilla*, *Urtica dioica*, *Taraxacum officinale*, *Valeriana officinalis* and *Quercus* sp. bark^54^ (numbered 502–507) were applied to the compost for the biodynamic plots (Table 3). After analysis of the composts the same amount of nitrogen equivalents were applied to every treatment^21^.

Nitrogen supply of the organic and the biodynamic treatments was ensured by soil cultivation and plowing in of the cover crop mixture in every second row shortly before full bloom. In the integrated plots a grass mixture of *Lolium perenne* and *Poa pratensis* was established as cover crop between rows. Every second row was plowed in shortly before bloom together with the cover crop of the organic and the biodynamic treatments. The integrated plots were amended with mineral fertilizers (50 kg N ha^−1^ on 06/26/10, one day after full-bloom, 25 kg N ha^−1^ on 07/05/12, six days after full-bloom and 24 kg N ha^−1^ on 06/16/14, ten days after full-bloom) to compensate for the nitrogen import in the organic and the biodynamic treatments that occurred due to plowing in the cover crop rich in legumes. In 2015 no mineral fertilizers were used in the integrated plots and no compost was added to any of the systems. Herbicides were generally used in the integrated treatment twice a year^21^. In the very dry season 2015 Glyfos (5 l ha^−1^) was used as herbicide in the integrated plots once on 04/21/15 (Supplementary Table 4). Pest management was conducted by applying systemic fungicides in the integrated plots and copper, sulfur and plant resistance improvers in the organic and the biodynamic plots, respectively (Supplementary Tables 8 and 9).

Both organic and biodynamic treatments were managed identically, except that biodynamic preparations were only applied to the biodynamic plots. Horn manure and horn silica, the biodynamic field spray preparations, were each applied three times a year^55^. Horn silica was applied at grapevine phenological stages shortly before full-bloom, at veraison and shortly before harvest and horn manure was applied once after harvest and twice in spring. The cow pat pit preparation was applied once a year in the growing season parallel to tillage in the biodynamic plots in the case that no compost was applied^21^.

### Sample collection

**Figure 8:**
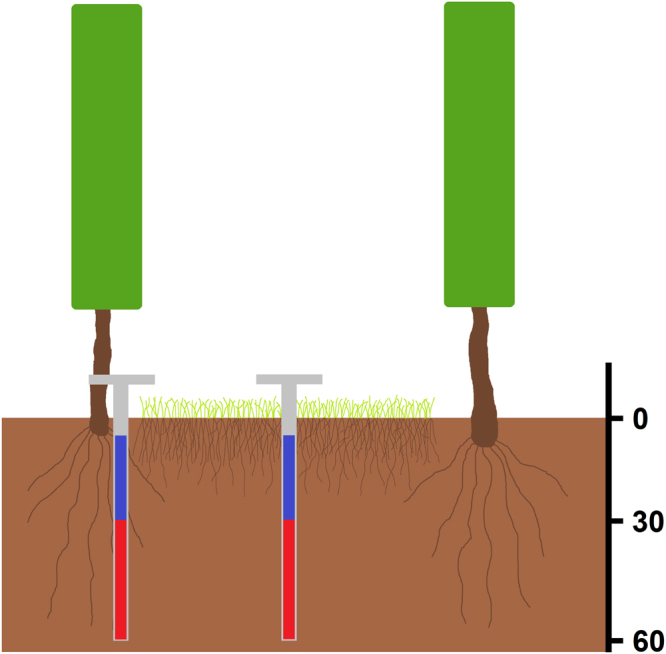
Soil sampling scheme.

Soil samples were collected in August 2015 as shown in Fig. 8 using a Pürckhauer soil sampler. Drill cores were taken in-row under cover cropping, and under-vine, respectively. The cores were divided into topsoil (5–30 cm) and subsoil (30–60 cm), and the first 5 cm under the surface were discarded to avoid major heterogeneities and point pollutions.

Within each block, mixed samples consisting of four different drillings were generated for each management regime, each position and each depth. Between each drilling, the drill stick and the equipment were washed with ethanol to avoid carryover effects. Soil samples were collected in sterile plastic bags, manually homogenized and immediately stored in a cooler. DNA extraction of all samples was conducted the same day.

### Soil Analysis

All physical and chemical soil parameters were determined at the Department of Soil Science and Plant Nutrition, Hochschule Geisenheim University, Germany, according to the VDLUFA database of methods^56^. Soil moisture content was determined gravimetrically after 48 h in a drying oven. For measuring pH levels, 20 g dried soil was extracted in 50 mL of 0.025 M CaCl_2_ solution. Potassium and phosphor were extracted from 5 g dried soil with 100 mL of CAL buffer (c(Ca-lactate) = 0.1 M, c(Ca-acetate) = 0.1 M, c(acetic acid) = 0.3 M) according to SCHUELLER^57^, and magnesium was extracted from 5 g dried soil with 50 mL of 0.0125 M calcium acetate buffer according to SCHACHTSCHABEL^57^. Phosphor was then measured by FIA (FIAstar 5000 Analyzer, Foss GmbH, Rellingen, Germany), potassium via AES (Elex 6361, Eppendorf AG, Hamburg, Germany) and magnesium by AAS (AAnalyst 200, PerkinElmer Inc., Waltham, MA, USA). Carbonate was determined according to SCHEIBLER^57^ based on the emergence of carbon dioxide after adding 2.8 M HCl in excess to 10 g dried soil. Total carbon and nitrogen were analyzed according to DUMAS^56^ by using a CHN analyzer (vario MAX CNS, Elementar Analysensysteme GmbH, Hanau, Germany). C_org_ and organic matter were computed subsequently. Total concentrations of heavy metals were extracted by aqua regia from 5 g of dried soil over 45 minutes in a microwave generator (MLS-1200 MEGA, MLS GmbH, Leutkirch, Germany), and measured via ICP-OES (ARCOS FHS12, Spectro Analytical Instruments GmbH, Kleve, Germany). Bioavailable heavy metal concentrations shared the same measurement technique, preceded by a CAT extraction (c(CaCl_2_) = 0.01 M, c(DTPA) = 0.002 M)^57^.

### DNA extraction, PCR and sequencing

DNA was extracted from 0.25 g of freshly homogenized soil using a PowerSoil DNA Isolation Kit (MoBio Laboratories Inc., Carlsbad, CA, USA), following the manufacturer’s protocol. The obtained DNA was stored at −80 °C until further processing. Spectrophotometry using a NanoDrop ND-8000 (ThermoFisher Scientific, Dreieich, Germany) of genomic template DNA proved consistent DNA concentrations in the extracts. For amplification of fungal ITS2 regions, a primer mix P5-5 N-ITS4 and P5-6 N-ITS4 (*forward*) together with P7-3 N-fITS7 and P7-4 N-fITS7 (*reverse*) was used according to Ihrmark *et al*.^58^. Amplification of bacterial 16S-rRNA genes utilized a primer mix P5-8 N-515 F and P5-7 N-515 F (*forward*) in combination with the primer mix P7-2 N-806r and P7-1 N-806r (*reverse*), as modified by Caporaso *et al*.^59^ (Table 4).

**Table 4:** Sequence overview of utilized primer.

primer name	primer sequence 5′-3′
P5–5N-ITS4	ACACTCTTTCCCTACACGACGCTCTTCCGATCTNNNNNTCCTCCGCTTATTGATATGC
P5–6N-ITS4	ACACTCTTTCCCTACACGACGCTCTTCCGATCTNNNNNNTCCTCCGCTTATTGATATGC
P7–3N-fITS7	GTGACTGGAGTTCAGACGTGTGCTCTTCCGATCTNNNGTGARTCATCGAATCTTTG
P7–4N-fITS7	GTGACTGGAGTTCAGACGTGTGCTCTTCCGATCTNNNNGTGARTCATCGAATCTTTG
P5–8N-515F	ACACTCTTTCCCTACACGACGCTCTTCCGATCTNNNNNNNNGTGCCAGCMGCCGCGGTAA
P5–7N-515F	ACACTCTTTCCCTACACGACGCTCTTCCGATCTNNNNNNNGTGCCAGCMGCCGCGGTAA
P7–2N-806r	GTGACTGGAGTTCAGACGTGTGCTCTTCCGATCTNNGGACTACHVGGGTWTCTAAT
P7–1N-806r	GTGACTGGAGTTCAGACGTGTGCTCTTCCGATCTNGGACTACHVGGGTWTCTAAT
P5-index	5′-AATGATACGGCGACCACCGAGATCTACACiiiiiiii ACACTCTTTCCCTACACGACGCTCTTCCGATC*T
P7-index	5′-CAAGCAGAAGACGGCATACGAGATiiiiiiii GTGACTGGAGTTCAGACGTGTGCTCTTCCGATC*T

Abbreviations according to IUPAC Ambiguity Code.

For polymerase chain reaction, 25 µl reactions were composed in triplicates, containing 12.5 µl of GoTaq Green Mastermix (Promega, Madison, USA), 1 μl of *forward*-primer mix (10 μM), 1 μl of *reverse*-primer mix (10 μM), 9.5 μl of nuclease-free water and 1 μl of template DNA. Amplification of fungal ITS2-rRNA followed cycler conditions: denaturation period over 5 min at 95 °C, followed by 32 cycles of 95 °C for 1 min, 55 °C for 1 min, 72 °C for 1 min 15 sec and a final elongation step at 72 °C for 10 min. For amplification of bacterial 16S-rRNA the cycler conditions were: denaturation period over 3 min at 94 °C, followed by 29 cycles of 94 °C for 45 sec, 50 °C for 1 min, 72 °C for 1 min 30 sec and a final elongation step at 72 °C for 10 min. PCR products were quality checked by separation on a 1.5% agarose gel. Triplicates were recovered from agarose gel, pooled and purified via gel extraction using the innuPREP Gel Extraction Kit (Analytik Jena, Jena, Germany) following the manufacturer’s protocol. PCR yield was then quantified on a spectrophotometer using Quant-iTTM PicoGreen® dsDNA reagent (Thermo Fisher Scientific Inc., Waltham, MA, USA) according to Ahn^60^. Based on their DNA concentrations, samples were pooled in a 1:1 ratio and diluted to a uniform concentration of 10 ng μl^−1^ by addition of TE buffer. Subsequently the PCR products were sequenced with an Illumina MiSeq at the Deep Sequencing Group of the Technical University Dresden. Briefly, purified PCR products with universal 5′ tails were subjected to a second PCR of 6–8 cycles using Phusion HF (NEB) and two indexing primers, the P5 and the P7 index primer (Table 2). After indexing PCR, the final libraries were purified (1 × XP Beads, Agencourt), equimolarily pooled and used for 2 × 300 bp paired end sequencing on a MiSeq System from Illumina resulting in approximately 20–25 million reads per flowcell.

### Raw data and bioinformatic analysis

Raw data (FASTQ files) were further processed using Geneious R9 software^61^. 5′ ends of all sequences were trimmed and adapters were removed. Forward and reverse reads were paired and subsequently separated into fungal and bacterial sequences with the help of their specific primer sequence. Paired reads were quality trimmed by BBDUK (*trim low quality*, *minimum quality* = *13*) and then merged via BBMerge (*merge rate* = *very high*). Sequence length fragments below 200 bp and above 450 bp were discarded. Using the Mothur software (Version 1.36.1)^62^ chimera were filtered by the *chimera*.*uchime* command, excluding 5,530 fungal and 69,330 bacterial sequences. The remaining data was clustered into *operational taxonomic units* (OTUs) based on 97% consensus utilizing CD-HIT-EST^63^. Finally, singletons, doubletons and tripletons were removed as they were considered as process-related artefacts.

In addition, a FASTA file with representative sequences was generated for fungal and bacterial OTUs. Fungal sequences were blasted via *Megablast* against an adapted database containing known full-length ITS from nr database, and bacterial sequences against the complete NCBI *Bacteria* nr database. For phylogenetic analyses, sequences of the *Megablast* hits given for the 30 most abundant fungal respectively bacterial OTUs were downloaded from NCBI. Subsequently, a *MAFFT* alignment was performed and a *Maximum likelihood* dendrogram was generated (substitution model = *General-time-reversible*, optimization by topology, branch length and substitution rate) including *Bootstrap* values based on 100 replicates.

### Statistics

Statistical analyses were performed using R^64^ and its user interface Rstudio^65^ as well as Geneious (version 9.1.2), und Past (version 2.17c).

Univariate analyses of variance were estimated by means of R packages *lme4*^66^ and *lmerTest*^67^ for generating mixed linear models, with management system, soil depth and sampling position as independent variables as well as a subsequent exclusion of random block effects. *Least Significant Difference* was performed as Post-hoc test using the R packages *lsmeans*^68^ and *multcomp*^69^, levels of significance were FDR adjusted. Species richness values were normalized previously.

Implementing the R packages *vegan*^70^, saturation levels of samples were calculated using rarefaction (for results, see Supplementary Figs 5 and 6). Furthermore, *permutational multivariante analyses of variance* (PERMANOVA) were conducted via the *adonis* command, based on Bray-Curtis distances with 999 permutations. Multivariate homogeneity of group dispersions was calculated and plotted as *principal coordinates analysis* (PCoA) using the *betadisper* command for the factors management and position, respectively. With *analysis of similarities* (ANOSIM) p-values for comparisons of community groups were computed. Finally, the *envfit* command was applied to check for correlations between community structures and soil parameters.

### Data availability

Sequence data are available in the short read archive (SRA) of the National Center for Biotechnology Information (NCBI), under accession numbers SRP107318. Raw data of soil analyses, fungal and bacterial OTU tables as well as their corresponding taxonomic *Megablast* hits can be reviewed in the Supplementary Dataset Files 1–5.

## Electronic supplementary material

Supplementary Information

Supplementary Dataset 1

Supplementary Dataset 2

Supplementary Dataset 3

Supplementary Dataset 4

Supplementary Dataset 5
