# Supplementary material for: Effects of different management regimes on microbial biodiversity in vineyard soils
Source: Sci Rep. 2018 Jun 20;8:9393. doi: 10.1038/s41598-018-27743-0 (PMC6010416; doi:10.1038/s41598-018-27743-0)
Supplement: Supplementary file 1 — Supplementary Information [file 41598_2018_27743_MOESM1_ESM.pdf]

Effects of different management regimes on microbial biodiversity in vineyard soils

Maximilian Hendgen[1,2*], Björn Hoppe[3,4], Johanna Döring[2], Matthias Friedel[2], Randolf Kauer[2], Matthias Frisch[5], Andreas Dahl[6], Harald Kellner[7]

[1] Department of Soil Science and Plant Nutrition, Hochschule Geisenheim University, Geisenheim, Germany
[2] Department of General and Organic Viticulture, Hochschule Geisenheim University, Geisenheim, Germany
[3] Department of Soil Ecology, Helmholtz Centre for Environmental Research – UFZ, Halle, Germany
[4] Institute for National and International Plant Health, Julius Kühn-Institute, Braunschweig, Germany
[5] Institute of Agronomy and Plant Breeding, Justus Liebig University, Gießen, Germany
[6] Deep Sequencing Group - Biotechnology Center Technische Universität Dresden, Germany
[7] Department of Bio- and Environmental Sciences, International Institute Zittau, Technische Universität Dresden, Germany

* Maximilian.Hendgen@hs-gm.de (MH)

Supplementary Information

Supplementary Table 1. Results of soil analyses

management	integrated				organic				biodynamic			
**position**	**in-row**		**under-vine**		**in-row**		**under-vine**		**in-row**		**under-vine**	
**depth (cm)**	**5-30**	**30-60**	**5-30**	**30-60**	**5-30**	**30-60**	**5-30**	**30-60**	**5-30**	**30-60**	**5-30**	**30-60**
**soil moisture content (%)**	7.5 ±0.50^{d}	8.2 ±0.61^{cd}	8.6 ±0.54^{bc}	9.5 ±0.50^{a}	8.6 ±0.74^{abc}	8.9±0.74^{abc}	8.4 ±0.38^{bc}	8.8 ±1.24^{abc}	8.5 ±0.65^{bc}	8.7 ±0.79^{abc}	9.3 ±0.23^{ab}	9.3 ±0.97^{ab}
**pH**	7.3 ±0.18^{bc}	7.4 ±0.13^{abc}	7.4 ±0.13^{abc}	7.4 ±0.15^{a}	7.3 ±0.19^{ab}	7.4 ±0.12^{ab}	7.3 ±0.22^{bc}	7.4 ±0.15^{ab}	7.4 ±0.05^{ab}	7.4 ±0.10^{ab}	7.3 ±0.05^{abc}	7.4 ±0.00^{ab}
**K (mg K_2O 100 g soil^{-1})**	35.5 ±9.32^{a}	29.0 ±7.26^{bc}	30.5 ±6.95^{b}	23.8 ±6.85^{de}	37.0 ±6.73^{a}	23.8 ±4.19^{de}	27.0 ±4.97^{bcd}	21.3 ±2.75^{e}	36.5 ±6.02^{a}	25.3 ±5.12^{cde}	36.3 ±1.89^{a}	24.3 ±2.63^{de}
**Mg (mg Mg 100 g soil^{-1})**	10.8 ±3.50^{bc}	12.3 ±2.75^{abc}	11.8 ±2.75^{bc}	13.0 ±2.58^{ab}	12.5 ±2.65^{abc}	15.3 ±2.50^{a}	12.5 ±1.91^{abc}	11.0 ±5.35^{bc}	10.0 ±1.41^{bc}	12.5 ±0.58^{abc}	10.8 ±1.50^{bc}	9.8 ±5.85^{c}
**P (mg P_2O_5 100 g soil^{-1})**	73.0 ±18.62^{ab}	79.5 ±5.92^{ab}	78.0 ±23.96^{ab}	72.5 ±17.75^{ab}	76.5 ±9.95^{ab}	71.8 ±5.50^{ab}	71.0 ±13.34^{b}	74.5 ±3.70^{ab}	80.0 ±14.17^{ab}	82.5 ±11.09^{ab}	86.5 ±9.47^{a}	80.8 ±10.34^{ab}
**soil organic matter (%)**	2.3 ±0.26^{a}	1.8 ±0.28^{cd}	2.1 ±0.05^{ab}	1.7 ±0.21^{cde}	2.1 ±0.18^{a}	1.7 ±0.16^{cde}	1.9 ±0.13^{bc}	1.6 ±0.17^{e}	2.1 ±0.17^{a}	1.6 ±0.23^{de}	2.1 ±0.12^{ab}	1.8 ±0.05^{cde}
**soil organic carbon (%)**	1.31 ±0.15^{a}	1.05 ±0.16^{cd}	1.21 ±0.03^{ab}	0.97 ±0.10^{cde}	1.21 ±0.10^{a}	0.98 ±0.09^{cde}	1.08 ±0.07^{bc}	0.90 ±0.12^{e}	1.22 ±0.09^{a}	0.95 ±0.12^{de}	1.19 ±0.07^{ab}	1.02 ±0.05^{cde}
**total nitrogen (%)**	0.10 ±0.02^{ab}	0.08 ±0.02^{cde}	0.09 ±0.02^{abc}	0.08 ±0.02^{de}	0.10 ±0.01^{a}	0.08 ±0.01^{de}	0.09 ±0.01^{bcd}	0.07 ±0.01^{e}	0.10 ±0.02^{a}	0.08 ±0.01^{de}	0.10 ±0.01^{a}	0.08 ±0.01^{de}
**C:N ratio**	14.3 ±3.30^{a}	13.0 ±1.41^{ab}	13.3 ±2.50^{ab}	13.5 ±4.51^{ab}	12.5 ±0.58^{ab}	13.0 ±1.41^{ab}	12.8 ±0.96^{ab}	12.3 ±0.50^{ab}	12.8 ±0.96^{ab}	12.5 ±2.38^{ab}	11.75 ±0.96^{b}	12.8 ±0.96^{ab}
**total Cu (mg kg soil^{-1})**	96.3 ±17.54^{a}	82.6 ±24.39^{a}	94.9 ±16.02^{a}	82.3 ±30.06^{a}	105.7 ±50.89^{a}	106.3 ±14.26^{a}	101.7 ±16.53^{a}	97.3 ±25.07^{a}	97.1 ±4.11^{a}	81.8 ±23.20^{a}	111.7 ±22.74^{a}	84.0 ±29.93^{a}
**total Fe (mg kg soil^{-1})**	22183 ±2478^{bc}	23043 ±1708^{abc}	21765 ±3012^{c}	22110 ±2709^{c}	23298 ±2266^{abc}	22395 ±1435^{abc}	22920 ±1994^{abc}	22453 ±1608^{abc}	23253 ±3396^{abc}	23468 ±3538^{abc}	24160 ±3514^{ab}	24285 ±3303^{a}
**total Mn (mg kg soil^{-1})**	620 ±26.1^{cd}	669 ±105.8^{abc}	623 ±35.2^{cd}	593 ±28.0^{d}	649 ±21.5^{abcd}	614 ±48.6^{cd}	637 ±35.6^{bcd}	616 ±62.2^{cd}	674 ±46.8^{abc}	718 ±63.1^{a}	700 ±41.0^{ab}	715 ±51.2^{a}
**total Ni (mg kg soil^{-1})**	25.7 ±2.91^{c}	27.6 ±3.21^{abc}	25.8 ±4.03^{c}	25.9 ±3.71^{c}	28.2 ±4.09^{abc}	27.3 ±4.23^{a}	27.2 ±4.21^{bc}	26.6 ±4.36^{bc}	28.4 ±4.92^{abc}	28.7 ±4.90^{abc}	30.1 ±5.54^{ab}	31.3 ±9.91^{a}
**total Zn (mg kg soil^{-1})**	93.9 ±10.76^{abc}	90.7 ±1.98^{bc}	101.2 ±18.85^{abc}	87.3 ±14.36^{c}	104.3 ±13.69^{abc}	89.9 ±12.04^{bc}	96.9 ±16.09^{abc}	94.6 ±20.52^{abc}	105.9 ±14.60^{abc}	109.3 ±35.05^{ab}	112.7 ±21.35^{a}	93.0 ±10.36^{abc}
**bioavailable Cu (mg kg soil^{-1})**	23.6 ±3.47^{abc}	19.5 ±3.56^{bcd}	24.4 ±8.60^{abc}	23.5 ±8.33^{abcd}	27.3 ±6.11^{a}	19.0 ±0.74^{cd}	24.2 ±4.30^{abc}	24.4 ±4.17^{abc}	21.3 ±6.09^{abcd}	17.5 ±3.88^{d}	25.1 ±7.27^{ab}	18.6 ±3.85^{cd}
**bioavailable Fe (mg kg soil^{-1})**	30.6 ±12.87^{ab}	25.6 ±10.11^{abcd}	28.9 ±11.44^{abc}	24.6 ±7.36^{abcd}	33.9 ±22.17^{a}	21.4 ±5.70^{bcd}	22.8 ±8.27^{bcd}	27.6 ±15.45^{abcd}	23.3 ±1.89^{abcd}	20.8 ±2.83^{bcd}	19.9 ±1.99^{cd}	18.1 ±1.94^{d}
**bioavailable Mn (mg kg soil^{-1})**	41.4 ±22.88^{ab}	30.2 ±13.20^{b}	34.5 ±21.04^{ab}	33.6 ±19.60^{ab}	57.2 ±61.42^{a}	25.6 ±9.09^{b}	26.3 ±9.35^{b}	42.9 ±38.55^{ab}	28.1 ±2.93^{b}	28.6 ±5.40^{b}	25.2 ±2.01^{b}	20.6 ±6.12^{b}
**bioavailable Zn (mg kg soil^{-1})**	12.7 ±1.25^{a}	11.1 ±2.74^{a}	14.5 ±3.46^{a}	12.0 ±2.94^{a}	14.8 ±2.39^{a}	10.5 ±1.71^{a}	12.1 ±1.49^{a}	12.1 ±2.58^{a}	13.8 ±2.51^{a}	15.7 ±13.00^{a}	14.5 ±3.51^{a}	10.4 ±1.90^{a}
**B (mg kg soil^{-1})**	0.56 ±0.07^{abc}	0.59 ±0.21^{abc}	0.52 ±0.10^{bc}	0.55 ±0.14^{abc}	0.50 ±0.19^{bc}	0.47 ±0.15^{bc}	0.40 ±0.11^{c}	0.49 ±0.12^{bc}	0.44 ±0.05^{bc}	0.59 ±0.12^{abc}	0.83 ±0.61^{a}	0.71 ±0.31^{ab}

Results as mean values ± standard deviation. Superscript letters correspond to significant differences (α = 0.05)

Supplementary Table 2. PERMANOVA of fungal species communities in topsoil.

	Df	SumsOfSqs	MeanSqs	F.Model	R2	Pr(>F)	
**management**	2	0.41671	0.20835	1.9347	0.13506	0.002	**
**position**	1	0.48042	0.48042	4.4609	0.15571	0.001	***
**management:position**	2	0.2497	0.12485	1.1593	0.08093	0.17	
**Residuals**	18	1.9385	0.10769		0.6283		
**Total**	23	3.08533			1		

Significance code: [.] < α = 0.1; [*] < α = 0.05; [**] < α = 0.01; [***] < α = 0.001

Supplementary Table 3. PERMANOVA of fungal species communities in subsoil.

	Df	SumsOfSqs	MeanSqs	F.Model	R2	Pr(>F)	
**management**	2	0.32053	0.16027	1.34623	0.11008	0.012	*
**position**	1	0.34131	0.34131	2.86703	0.11721	0.001	***
**management:position**	2	0.22621	0.11311	0.95009	0.07769	0.414	
**Residuals**	17	2.02381	0.11905		0.69502		
**Total**	22	2.91187			1		

Significance code: [.] < α = 0.1; [*] < α = 0.05; [**] < α = 0.01; [***] < α = 0.001

Supplementary Table 4. PERMANOVA of fungal species communities in topsoil in-row.

	Df	SumsOfSqs	MeanSqs	F.Model	R2	Pr(>F)	
**management**	2	0.36536	0.18268	1.6779	0.2716	0.005	**
**Residuals**	9	0.97985	0.10887		0.7284		
**Total**	11	1.34521			1		

Significance code: [.] < α = 0.1; [*] < α = 0.05; [**] < α = 0.01; [***] < α = 0.001

Supplementary Table 5. PERMANOVA of fungal species communities in topsoil undervine.

	Df	SumsOfSqs	MeanSqs	F.Model	R2	Pr(>F)	
**management**	2	0.30105	0.15052	1.4131	0.23898	0.076	.
**Residuals**	9	0.95865	0.10652		0.76102		
**Total**	11	1.2597			1		

Significance code: [.] < α = 0.1; [*] < α = 0.05; [**] < α = 0.01; [***] < α = 0.001

Supplementary Table 6. Correlation analysis between fungal community and soil parameters.

	NMDS1	NMDS2	r2	Pr(>r)	
**soil_moisture**	0.9197	0.39261	0.2398	0.002	**
**pH**	0.84741	-0.53094	0.2014	0.001	***
**P**	0.99422	-0.1074	0.0261	0.447	
**K**	-0.9461	0.32389	0.4086	0.001	***
**Mg**	-0.28325	-0.95905	0.0448	0.403	
**organic carbon**	-0.98084	0.19482	0.5214	0.001	***
**N**	-0.89264	0.45076	0.3063	0.001	***
**C:N**	-0.66766	-0.74447	0.0385	0.241	
**Mn_total**	0.68427	-0.72923	0.0164	0.674	
**Fe_total**	0.83153	0.55548	0.0553	0.164	
**Cu_total**	0.0801	0.99679	0.04	0.457	
**Zn_total**	0.35337	0.93548	0.0177	0.669	
**Ni_total**	0.92367	0.38318	0.0684	0.227	
**Mn_bioav**	-0.98608	0.16624	0.1193	0.046	*
**Fe_bioav**	-0.99633	0.08555	0.1717	0.01	**
**Cu_bioav**	-0.31004	0.95072	0.0906	0.09	.
**Zn_bioav**	-0.96414	0.2654	0.0133	0.693	
**B**	0.97107	0.23879	0.0015	0.967	

Stress of the underlying two-dimensional NMDS based on Bray-Curtis distances was 0.21.

Supplementary Table 7. Correlation analysis between bacterial community and soil parameters.

	NMDS1	NMDS2	r2	Pr(>r)	
**soil_moisture**	0.9962	-0.0871	0.102	0.051	.
**pH**	0.78453	0.62009	0.4885	0.026	*
**P**	0.57636	-0.8172	0.0255	0.677	
**K**	-0.99285	-0.11933	0.4186	0.001	***
**Mg**	-0.20717	-0.9783	0.3704	0.046	*
**organic_carbon**	-0.93595	0.35213	0.5632	0.001	***
**N**	-0.99497	-0.10015	0.3615	0.002	**
**C:N**	-0.04692	0.9989	0.0904	0.315	
**Mn_total**	0.03222	0.99948	0.1821	0.014	*
**Fe_total**	0.57548	0.81782	0.0444	0.461	
**Cu_total**	0.99955	0.0299	0.0386	0.306	
**Zn_total**	0.17294	0.98493	0.2083	0.017	*
**Ni_total**	0.70883	0.70538	0.1288	0.404	
**Mn_bioav**	-0.67922	0.73394	0.07	0.075	.
**Fe_bioav**	-0.69154	-0.72234	0.3579	0.063	.
**Cu_bioav**	-0.73283	-0.68041	0.4857	0.01	**
**Zn_bioav**	-0.79198	0.61054	0.0383	0.324	
**B**	-0.21804	-0.97594	0.0467	0.668	

Stress of the underlying two-dimensional NMDS based on Bray-Curtis distances was 0.15.

Supplementary Table 8. Overview of integrated pest management in 2015.

#	date	plant protection agent	quantity	unit
1	04/21/15	Glyfos (under-vine)	5	l ha^{-1}
2	05/18/15	Delan WG	0.3	kg ha^{-1}
		wettable sulfur	3.6	kg ha^{-1}
3	06/01/15	Delan WG	0.3	kg ha^{-1}
		Vivando	0.16	l ha^{-1}
4	06/16/15	Delan WG	0.5	l ha^{-1}
		Veriphos	2.5	l ha^{-1}
		Collis	0.4	l ha^{-1}
5	06/29/15	Enervin	3	kg ha^{-1}
		Talendo	0.3	l ha^{-1}
6	07/13/15	Folpan	1.6	kg ha^{-1}
		Luna Experience	0.4375	l ha^{-1}
7	07/09/15	Teldor (in bunch zone)	0.6	kg ha^{-1}
8	07/27/15	Mildicut	4	l ha^{-1}
		Systhane	0.24	l ha^{-1}
		Steward	0.2	kg ha^{-1}
9	08/10/15	Mildicut	4	l ha^{-1}
		Systhane	0.24	l ha^{-1}
		Steward	0.2	kg ha^{-1}
10	08/27/15	Switch (in bunch zone)	0.64	kg ha^{-1}

Supplementary Table 9. Overview of organic and biodynamic pest management in 2015

#	date	plant protection agent	quantity	unit	amount of copper [g ha^{-1}]
1	05/18/15	wettable sulfur	3.6	kg ha^{-1}	
		Funguran Progress	0.29	kg ha^{-1}	100
2	06/01/15	wettable sulfur	3.6	kg ha^{-1}	
		Funguran Progress	0.29	l ha^{-1}	100
3	06.12.15	wettable sulfur	4.8	kg ha^{-1}	
		Funguran Progress	0.29	l ha^{-1}	100
4	06/17/15	wettable sulfur	2.4	kg ha^{-1}	
		Funguran Progress	0.86	l ha^{-1}	300
5	06/26/15	wettable sulfur	2.4	kg ha^{-1}	
		Funguran Progress	0.86	kg ha^{-1}	300
6	07/03/15	wettable sulfur	3.2	kg ha^{-1}	
		Funguran Progress	0.57	kg ha^{-1}	200
		Vitisan	4	kg ha^{-1}	
7	07/13/15	wettable sulfur	3.2	kg ha^{-1}	
		Funguran Progress	0.57	kg ha^{-1}	200
		Vitisan	4	kg ha^{-1}	
8	07/22/15	wettable sulfur	3.2	kg ha^{-1}	
		Funguran Progress	0.57	kg ha^{-1}	200
		Vitisan	4	kg ha^{-1}	
		PrevB2	1.2	l ha^{-1}	
9	07/31/15	wettable sulfur	3.2	kg ha^{-1}	
		Funguran Progress	0.57	kg ha^{-1}	200
		Vitisan	4	kg ha^{-1}	
		PrevB2	1.5	l ha^{-1}	
10	08/11/15	Funguran Progress	0.57	l ha^{-1}	200
		Vitisan	5	kg ha^{-1}	
		PrevB2	1.5	l ha^{-1}	

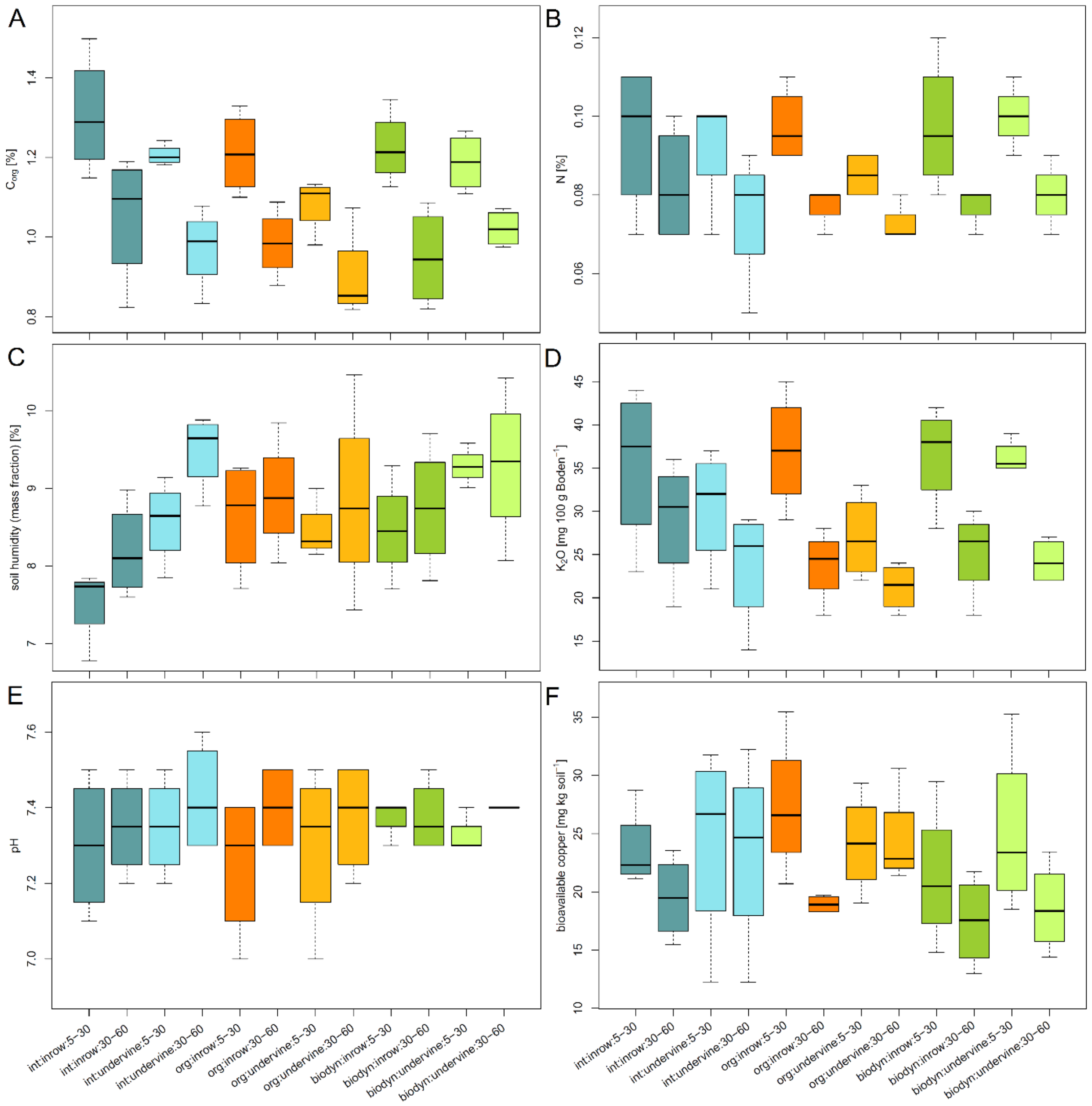

Supplementary Fig 1. Results of (A) organic carbon, (B) nitrogen, (C) soil humidity, (D) potassium, (E) pH and (F) bioavailable copper by management, position and depth.

Levels of significance:

C_{org}: p (management) < 0.022, p (position) < 0.024, p (depth) < 2.42E-10, no interaction effects

Nitrogen: p (management) < 0.196, p (position) < 0.141, p (depth) < 7.74E-8, no interaction effects

Humidity: p (management) < 0.085, p (position) < 0.003, p (depth) < 0.024, p (treatment:position) < 0.020

Potassium: p (management) < 0.019, p (position) < 1.48E-4, p (depth) < 2.02E-11, p (treatment:position) < 0.046

pH: p (management) < 0.390, p (position) < 0.359, p (depth) < 0.003, no interaction effects

bioavailable copper: p (management) < 0.126, p (position) < 0.111, p (depth) < 0.003, no interaction effects

(n = 4)

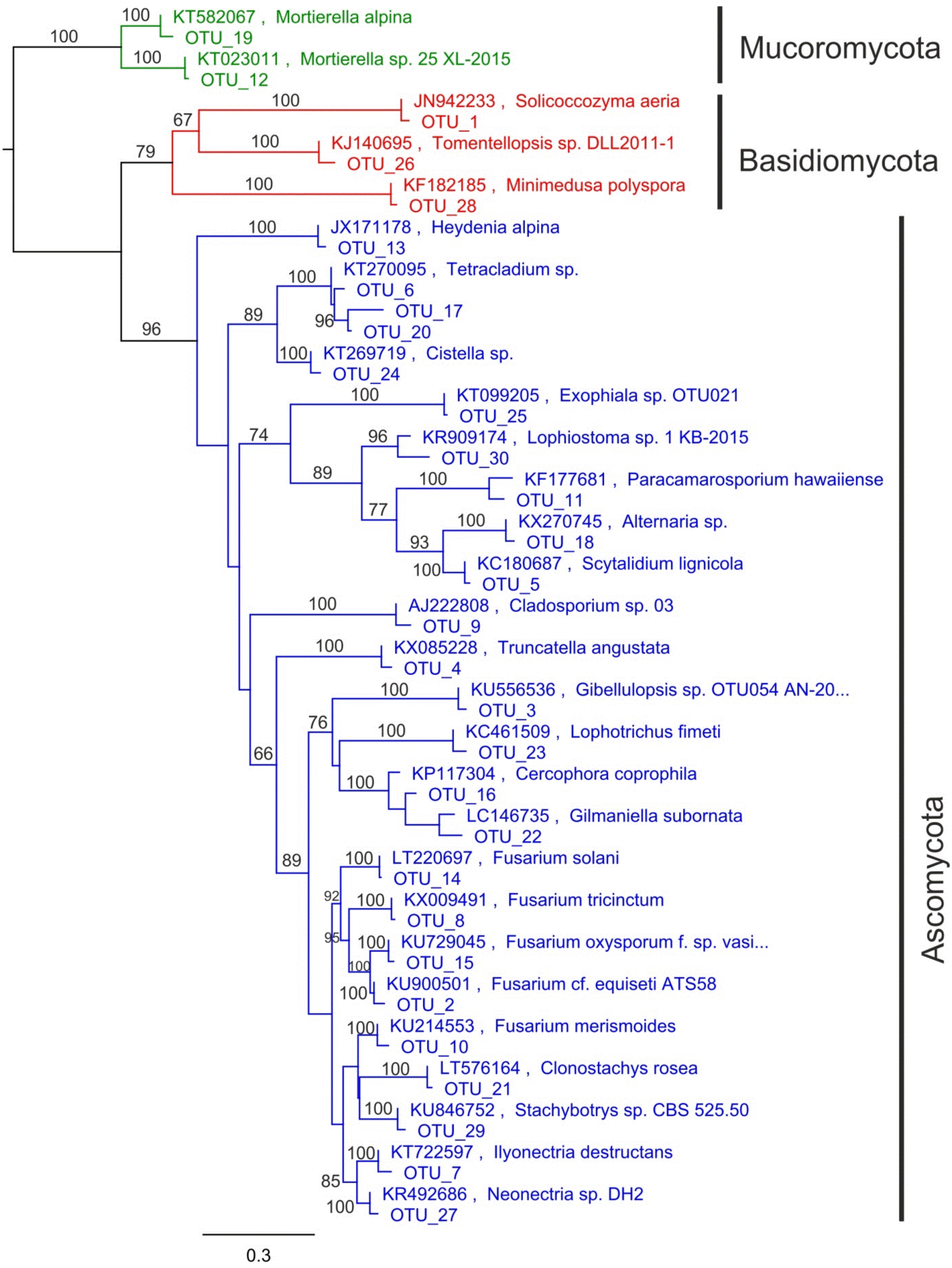

Supplementary Fig 2. Maximum-Likelihood dendrogram of fungal top-30 OTU's plus database hits. Bootstrap values on the branches are based on 100 permutations.
green: Mucoromycotina, red: Basidiomycota, blue: Ascomycota

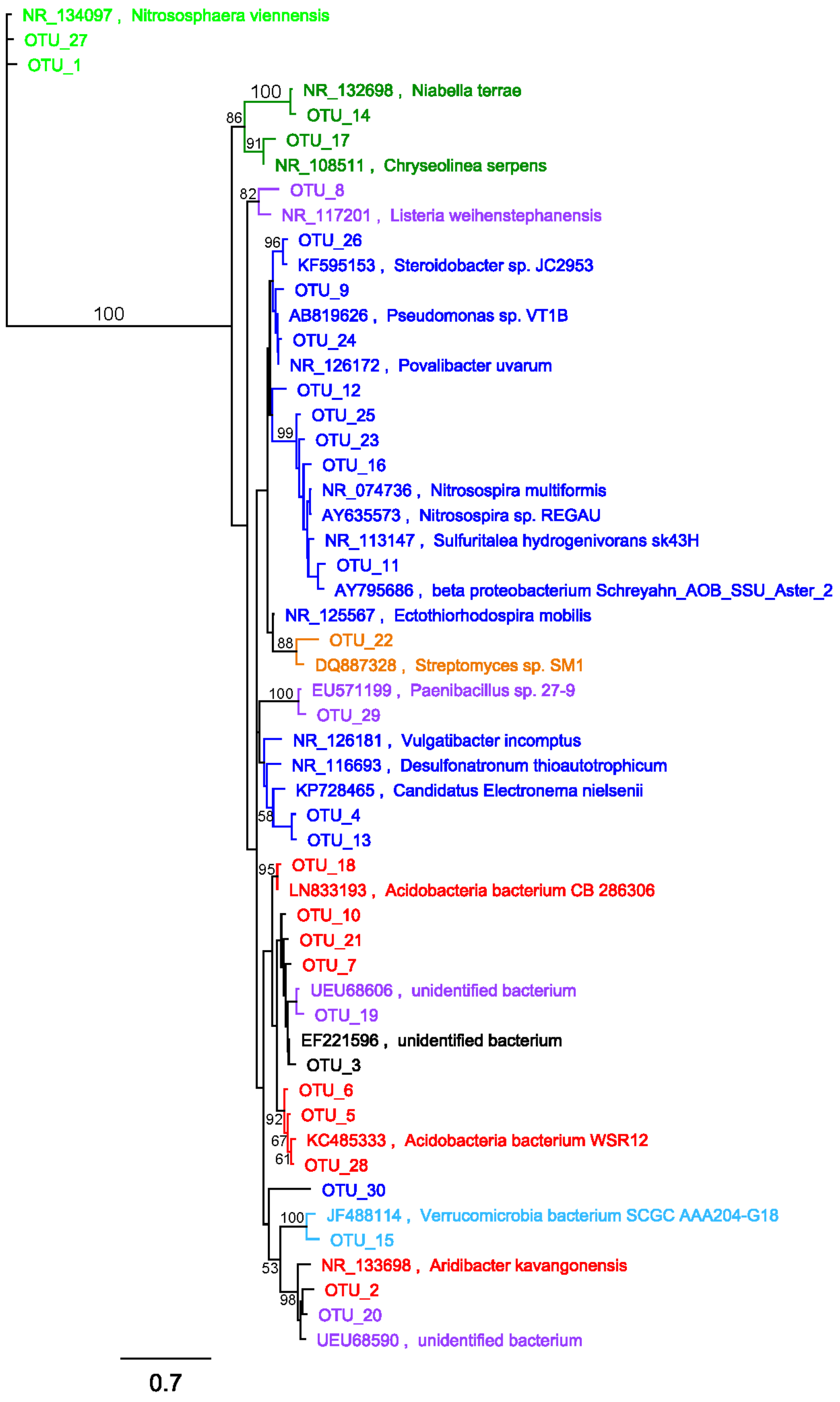

Supplementary Fig 3. Maximum-Likelihood dendrogram of bacterial top-30 OTU's plus database hits. Bootstrap values on the branches are based on 100 permutations.

light green: Archaea,red: Acidobacteria, orange: Actinobacteria, purple: Firmicutes, dark green: Bacteroidetes, light blue: Verrucomicrobia, dark blue: Proteobacteria, black: non-allocated

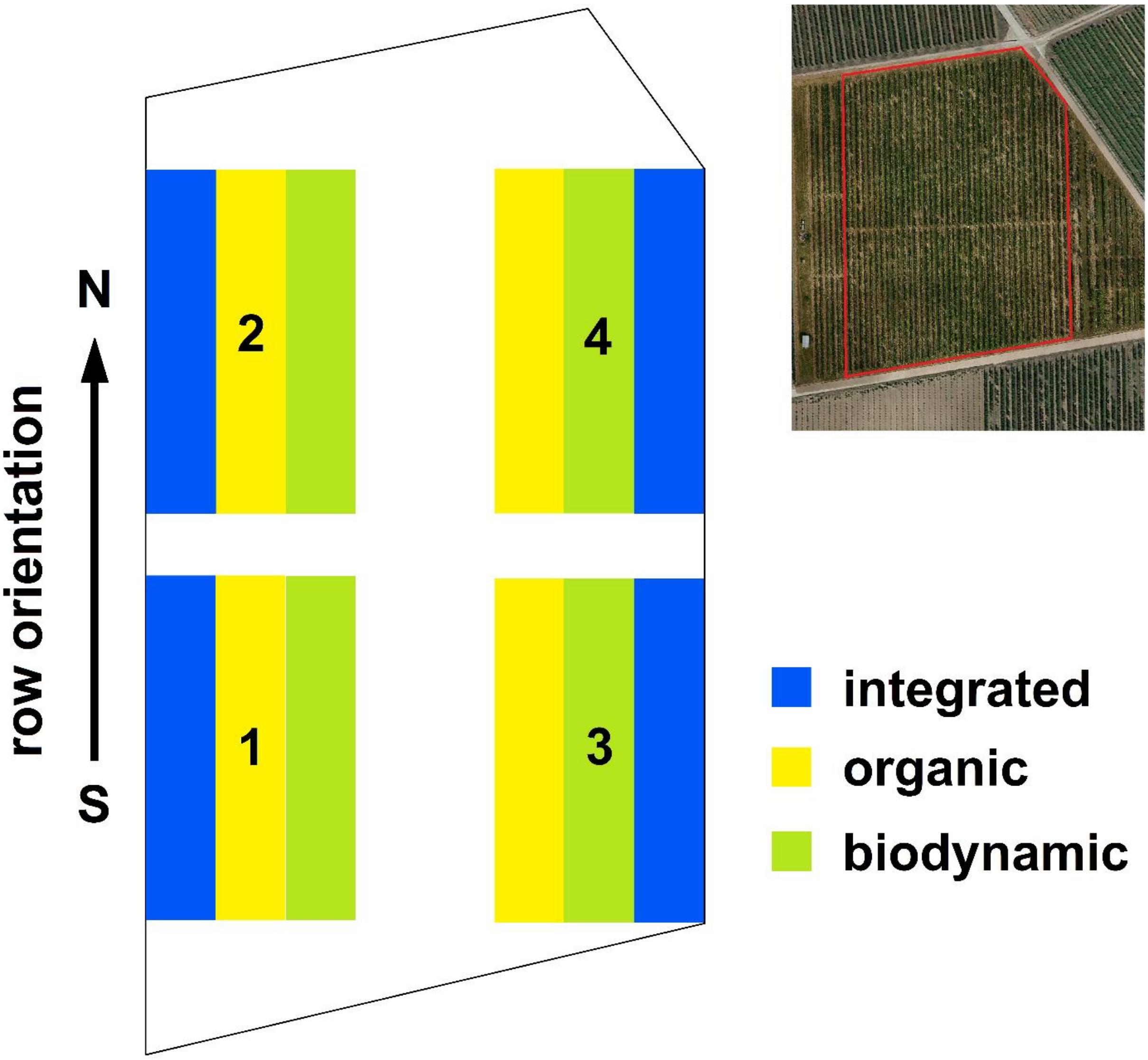

Supplementary Fig 4. Field plan of the experimental site.
The blocks are numbered 1 to 4.

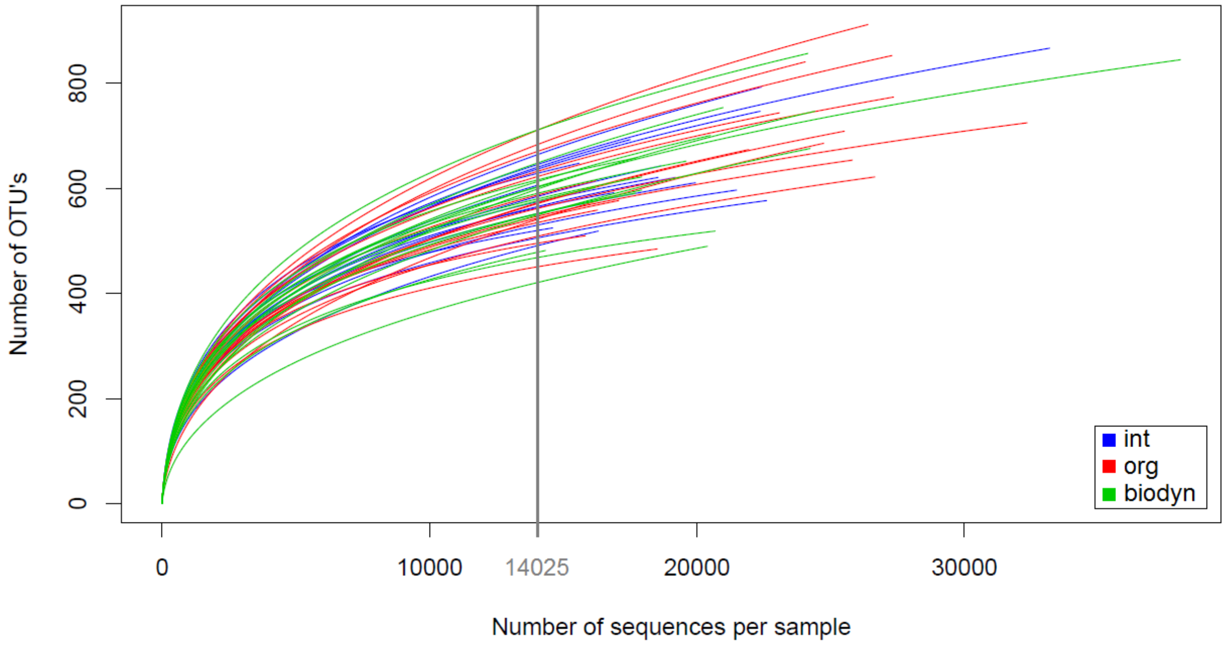

Supplementary Fig 5. Fungal rarefaction curves, based on raw data consisting of 992,075 sequences and 5,176 OTU's.
The grey line at 14025 sequences illustrates the limiting sample size for subsequent normalization.

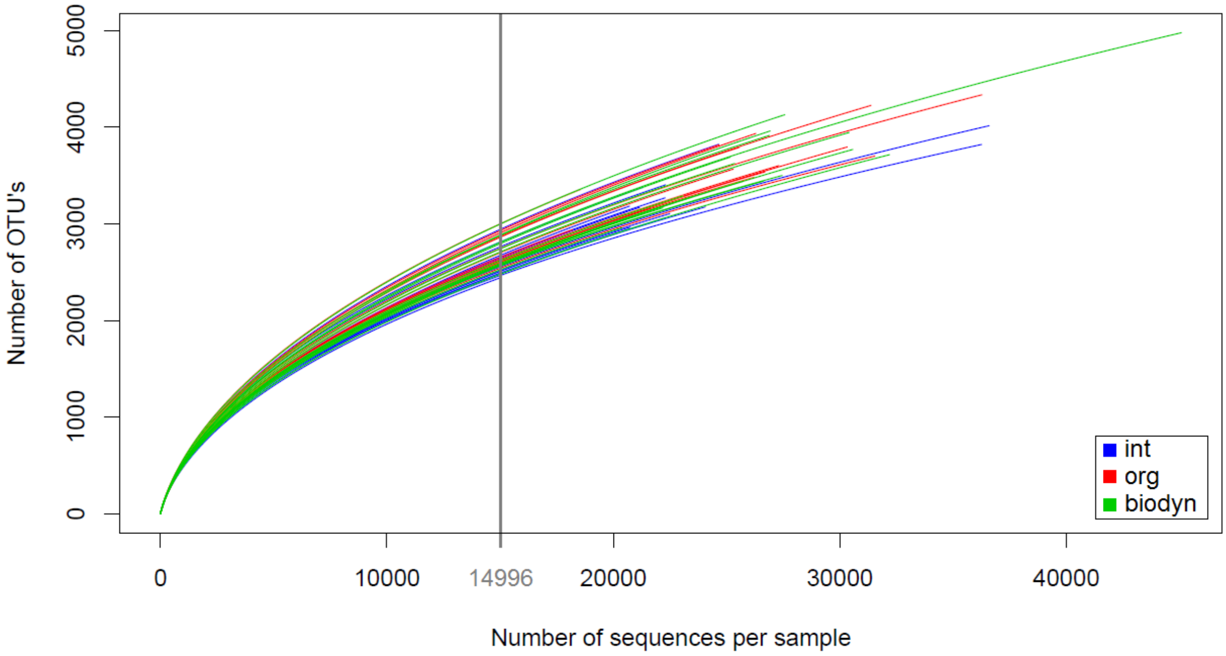

Supplementary Fig 6. Bacterial rarefaction curves, based on raw data consisting of 1,219,717 sequences and 27,516 OTU's.
The grey line at 14,025 sequences illustrates the limiting sample size for subsequent normalization.
